# Additional Exertion, Unsupported Assertions, and Hyperhydration Confound Rhabdomyolysis Case Study

**DOI:** 10.1093/milmed/usy237

**Published:** 2018-10-03

**Authors:** Russell Greene, Derek Fields

**Affiliations:** CrossFit Inc., 1250 Connecticut Ave NW Ste 700, Washington, DC

## Additional Exertion, Unsupported Assertions, and Hyperhydration Confound Rhabdomyolysis Case Study

I am writing to address Military Medicine’s recent article, “A Cluster of Exertional Rhabdomyolysis Cases in a ROTC Program Engaged in an Extreme Exercise Program,” by Raleigh et al. This piece requires correction. It makes unsubstantiated claims and omits evidence that would have complicated the cluster’s etiology and cast doubt upon its own conclusions. Without addressing the issues identified below, it is likely the article will be inaccurately cited as scientific evidence that the workout “Murph” and CrossFit training itself cause rhabdomyolysis.

The authors attribute a cluster of rhabdomyolysis cases solely to “a 1-mile run, 100 pull-ups, 200 push-ups, 300 squats, and another 1-mile run.” This workout is known as “Murph,” in honor of fallen Navy SEAL Lt. Michael P. Murphy, who created and performed it. This attribution is highly misleading. The authors ignore two other significant factors: the additional training the subjects performed in the days preceding the workout and the unit’s decision to hyperhydrate prior to exercise.

The Democrat and Chronicle coverage of the rhabdomyolysis cluster informs us, “the training in question took place over several days” and therefore the cluster “wasn’t all related to ‘The Murph’.”^1^ The newspaper cites the spokesman of The College at Brockport for this information.

Raleigh et al do not bother to ascertain what this other training consisted of, nor do they mention it at all. Hence, their attempt to place blame for the rhabdomyolysis cluster squarely on this single workout is highly misleading. To what extent did the other training sessions contribute to or cause these cases of rhabdomyolysis? Did they consist of novel training stimuli, abnormally high training volume, large repetitions of eccentric exercise or other known risk factors for rhabdomyolysis?^2^ Would the cluster have occurred had the subjects performed “Murph” absent any other training? The study does not answer these important questions, consider these factors, or even inform readers that there is any uncertainty at all regarding etiology.

The unit’s hyperhydration is also concerning. A College at Brockport official involved in the investigation informed my colleague Russell Berger that the unit aggressively hyperhydrated prior to physical training, in some cases consuming “multiple liters” of Gatorade and water beforehand. Such aggressive drinking is the primary cause of exercise-associated hyponatremia,^3^ itself “a likely etiological factor” behind exertional rhabdomyolysis.^4,5^

Did Raleigh et al fail to research the circumstances surrounding their subjects’ rhabdomyolysis? Or did they choose to disregard these facts? Had they interviewed school officials or even read local press coverage, the authors would have quickly discerned that much more than Murph was at play here and thus the article’s conclusions are, at best, misleading.

The authors furthermore allege, “The incidence of ER in the U.S. Military population appears to be on the rise, up 17.5% from 2014 to 2015… The popularity of extreme conditioning programs (ECPs) may play a role.” Their first source does demonstrate an increased incidence, but it does not give any indication of what caused that increase.^6^ To support their speculation that “extreme conditioning programs … may play a role” in this trend in the U.S. military, the authors strangely cite a study on civilians admitted to hospitals in Australia.^7^ The authors work for the U.S. military, but they do not cite a single source on this population to support the idea that what they label ECPs are driving an increase in incidences of rhabdomyolysis. As long as they are considering civilian hospitals, it might have been more appropriate to consider a 2016 study from an American hospital where spinning was the largest single cause of rhabdomyolysis, responsible for 14 of 29 total cases.^8^

The authors also neglect to cite a single source to support their allegation that, “it is clear that complications from ECPs are real, rising, and result in significant injury among U.S. service members.” The authors should share any evidence they have to support this claim with readers.

In the conclusion, they allege, “ER clusters appear to be on the rise, and both college and high school outbreaks have been reported as ECPs are adopted into training programs.” No source substantiates this claim, either. And the paper’s earlier review of college and high school athlete rhabdomyolysis included movements such as biceps curls and triangle push-ups, unintentionally rebutting any supposed association with CrossFit or other similar methodologies.

Finally, the authors conclude, “Functional high-intensity exercise programs should be used cautiously in vulnerable populations, in particular, basic trainees and cadets, and ECPs are not recommended.” This cluster of rhabdomyolysis cases does not support their conclusion. Instead, an accurate conclusion would have been, “It is not recommended to perform ECPs in addition to unknown quantities of additional physical training and in the presence of extreme hyperhydration of Gatorade and water.” Such a conclusion, however, would have required warning against hyperhydration and cautioning against doing too much, too quickly of any physical exercise, rather than blaming a single workout or methodology.
